# State-space reduction and equivalence class sampling for a molecular self-assembly model

**DOI:** 10.1098/rsos.150681

**Published:** 2016-07-20

**Authors:** Daniel M. Packwood, Patrick Han, Taro Hitosugi

**Affiliations:** 1 Advanced Institute for Materials Research (AIMR), Tohoku University, Sendai 980-8577, Japan; 2 Japan Science and Technology Agency (PRESTO), Kawaguchi, Saitama 332-0012, Japan; 3 California Nanosystems Institute, University of California, Los Angeles, CA 90095, USA; 4 Department of Chemistry and Biochemistry, University of California, Los Angeles, CA 90095, USA; 5 Department of Materials Science and Engineering, University of California, Los Angeles, CA 90095, USA; 6 Department of Applied Chemistry, Graduate School of Science and Engineering, Tokyo Institute of Technology, Tokyo 152-8352, Japan

**Keywords:** model reduction, Markov chain Monte Carlo, self-assembly

## Abstract

Direct simulation of a model with a large state space will generate enormous volumes of data, much of which is not relevant to the questions under study. In this paper, we consider a molecular self-assembly model as a typical example of a large state-space model, and present a method for selectively retrieving ‘target information’ from this model. This method partitions the state space into equivalence classes, as identified by an appropriate equivalence relation. The set of equivalence classes *H*, which serves as a reduced state space, contains none of the superfluous information of the original model. After construction and characterization of a Markov chain with state space *H*, the target information is efficiently retrieved via Markov chain Monte Carlo sampling. This approach represents a new breed of simulation techniques which are highly optimized for studying molecular self-assembly and, moreover, serves as a valuable guideline for analysis of other large state-space models.

## 1. Introduction

The oft-cited quote ‘We are drowning in information but starved for knowledge’ implies that information has become so abundant that it is nearly impossible to draw useful conclusions from it. The availability of affordable, high-performance computational resources allows for the simulation of sophisticated models with enormous state spaces. A researcher can, therefore, generate huge volumes of data with relatively little effort. On the other hand, the ‘target information’ desired by a researcher may be so deeply embedded in this dataset that it cannot be isolated without the use of elaborate statistical analysis. We will refer to these situations as *large state-space problems*. Techniques that efficiently recover target information from such large datasets are subject to intense research, and will remain of high priority so long as our hunger for facts and figures remains unsatisfied.

**Figure 1.**
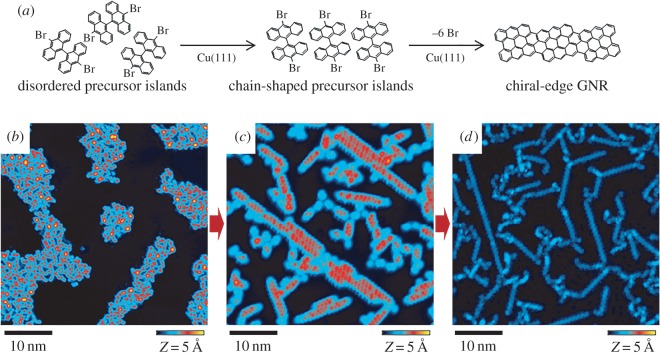
Formation of graphene by molecular self-assembly. (*a*) Self-assembly process represented by chemical formulae. (*b*–*d*) Scanning tunnelling microscopy images showing each step of the molecular self-assembly process.

As a case in point, we consider the phenomenon of molecular self-assembly, in which molecules deposited on a surface spontaneously assemble into highly ordered structures [1–3]. Figure 1*a* illustrates the fabrication of graphene by self-assembly of molecules on a copper surface [4]. Upon heating, the molecules assemble into chain-shaped ‘islands’ (figure 1*b,c*), and on further heating a chemical reaction occurs between molecules, resulting in graphene (figure 1*d*). Island formation is a necessary ‘stepping stone’ for graphene formation, and one goal of this experiment is to identify conditions (temperature, type of molecule and type of surface) that support island formation. However, this experiment also yields considerable additional information, including the positions of the islands on the surface, the distances between the islands and so on. This information does not help us to identify island-forming conditions, although it may be interesting in other contexts. We are faced with the following problem: build a model for the molecular self-assembly process and then estimate the probability of forming particular islands (the ‘target information’) *without* estimating any other properties of the model. Such a model should possess a very large state space in order to incorporate every conceivable outcome of the experiment in figure 1. Molecular self-assembly, therefore, provides an example of a large state-space problem and is of sufficient scientific importance to warrant attention.

Numerous models and methods have been employed in the chemistry and physics literature to study the molecular self-assembly process. Some recent reports have described molecular dynamics simulations [5–10], density functional theory [11], kinetic Monte Carlo simulations [12], stochastic models [13], graph theory [14] and mean-field models [15], to name a few. However, the possibility of studying self-assembly by selective retrieval of target information as described above has not been considered in this literature. In fact, the need for such a study seems to be particularly acute for the case of molecular self-assembly. For example, while molecular dynamics simulations should eventually yield reliable statistics on island formation, the simulation times required for the entire state space to be sufficiently sampled are inaccessible with modern computational power. The enormous state space of typical molecular self-assembly models, therefore, provides a barrier to both data collection *and* data analysis. Specialized mathematical techniques that can astutely access the target information embedded in a molecular self-assembly model would, therefore, provide a new generation tools for studying molecular self-assembly. Moreover, such techniques would provide useful guidelines for developing similar methods for other types of large state-space problems.

In this paper, we present the *equivalence class sampling* (ECS) technique for acquiring target information from a simple molecular self-assembly model. In the ECS technique, the state space of the model is partitioned into equivalence classes via an appropriate equivalence relation. Then, Markov chain Monte Carlo (MCMC) sampling [16] is performed on the *reduced state space H*, where *H* is the collection of equivalence classes. In this paper, two states belong to the same equivalence class if and only if they differ only in the position or orientation of their islands on the surface (precise details are provided in the main text). Recall that the target information for molecular self-assembly relates to the ‘kinds of islands’ that form under a given set of conditions. In ECS, all model configurations that contain the ‘same kinds of islands’ are placed into the same equivalence class. The reduced state space *H* therefore lacks all information unwanted by the researcher, such as the distance between islands or positions of islands in the surface. From a physical point of view, it can be shown that ECS directly samples the model properties with respect to a free energy, which is most desirable for any method which calculates equilibrium (stationary) physical properties. The price of these benefits is that ECS (as developed in this paper) requires a ‘low-coverage approximation’, in which the density of molecules on the surface is very small. However, such conditions are typical in experiments which study island formation via molecular self-assembly. Molecular self-assembly processes occurring under high-coverage conditions tend to produce ‘two-dimensional crystals’ rather than molecular islands [8,9], and are, therefore, not concerned with island formation. The ECS concept has been explored for characterization of graphical models of statistical dependence (Bayesian networks) [17–23]. However, the results obtained in these studies do not seem to directly apply to the molecular self-assembly problem, and new developments of the ECS technique are presented here. In particular, we develop and characterize the *extension–reduction chain*, a Markov chain with state space *H*, which in turn is used to generate proposals in the MCMC part (specifically, for a Metropolis–Hastings (MH) algorithm) of the ECS technique.

This paper is organized as follows. In §2, we introduce the self-assembly model used in this study, and in §3, we introduce the ECS approach for this problem. In §4, we present some simulation results, and in §5, we draw conclusions.

## 2. Chiral block assembly model

**Figure 2.**
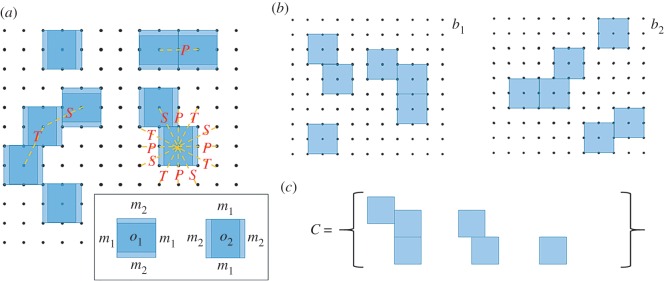
(*a*) Diagram of the chiral block assembly model (see text). (*b*) Pair of rotationally isomorphic states. (*c*) Canonical representation for the equivalence class containing the states in (*b*).

The chiral block assembly (CBA) model is a simple model for the molecular self-assembly process described in figure 1. Consider a finite square lattice **L**_*d*_ of dimension *d *×* d*, where *d* is an integer. *N blocks* reside on the lattice (figure 2*a*), where *N* is a fixed and given integer. A block is a square subset of the lattice that contains 3 × 3 points. The centre-most point of the block is called the *coordinate* of the block. The remaining eight points belong to the *perimeter* of the block. The perimeters of each block are decomposed as follows. Let *t* be an arbitrary block and let *F* denote the perimeter of *t*. Let *F*_1_ (respectively, *F*_2_, *F*_3_, *F*_4_) be the three points in *t* that lie above (respectively, to the left of, below, to the right of) the coordinate of *t*. The sets *F*_1_, *F*_2_, *F*_3_ and *F*_4_ are called *faces*, and *F* is the union of its faces. The block *t* is said to have one of two types of *orientations*, *o*_1_ and *o*_2_, and two *face types*, *m*_1_ and *m*_2_. If *t* has orientation *o*_1_, then faces *F*_1_ and *F*_3_ are of type *m*_1_, and faces *F*_2_ and *F*_4_ are of type *m*_2_. If *t* has orientation *o*_2_, then faces *F*_1_ and *F*_3_ are of type *m*_2_ and faces *F*_2_ and *F*_4_ are of type *m*_1_. Two blocks are said to *touch at their faces* if the intersection of their faces contains more than one point. In this case, the two blocks are said to be *neighbours*. Note that this definition excludes cases where two blocks only share a ‘corner’. Two blocks are said to *overlap* if their coordinates are contained in their intersections. The blocks may be arranged on the lattice in any way and can touch at their faces, but no two blocks may overlap.

To define an interaction energy between neighbouring blocks (pairs of blocks that touch at their faces), we first classify the ways in which neighbours can be *aligned* with respect to each other. These alignments are referred as *P*, *S* and *T* and the rules for assigning the alignments are described in figure 2*a*. Note that the rules or assigning alignments are independent of the orientation of the block. For each pair of neighbouring blocks *i* and *j*, we assign an *interaction energy* $\varepsilon_{r,m_{i},m_{j}}$, where *r* is the alignment of blocks *i* and *j* (*S*, *P* or *T*), and *m*_*i*_ (*m*_*j*_) is the face of block *i* (block *j*) which is touching the face of block *j* (block *i*). The condition $\varepsilon_{r,m_{i},m_{j}}=\varepsilon_{r,m_{j},m_{i}}$ is enforced. The collection of these interaction energies is called the *force field*. The adjective ‘chiral’ refers to the fact that the alignment between any two neighbours does not possess mirror symmetry. This feature occurs in many real cases of molecular self-assembly on metal surfaces [4,24]. The CBA model is a special case of Winfree's tile assembly model [25–27].

Without further mention, we will assume periodic boundary conditions on the CBA model. This is achieved by replacing the lattice **L**_*d*_ with the lattice **L**_*d*_ _+_ _1_ = *X*_*d*_ _+_ _1_ × *Y*_*d*_ _+_ _1_, where *X*_*d*_ _+_ _1_ = *Y*_*d*_ _+_ _1_ = {1, 2, … , *d*, *d* + 1}, and then, imposing that the lattice points 1 and *d* + 1 in *X*_*d*_ _+_ _1_ are identical and that lattice points 1 and *d* + 1 in *Y*_*d*_ _+_ _1_ are identical. This results in the torus **T**_*d*_. The nomenclature *lattice* and *dimension* will be taken to mean the set *X*_*d*_ × *Y*_*d*_ and the integer *d*, respectively.

A *configuration* is any choice of block positions and orientations that satisfies the conditions for the CBA model (no overlap of blocks). Let Ω be the collection of all configurations. For each σ∈Ω, we assign the probability

2.1 $$\pi (\sigma )\propto e^{-\varepsilon (\sigma )/k_{B}T_{0}},$$

where *k*_B_ and *T*_0_ are positive constants (the Boltzmann constant and temperature, respectively) and *ε*(*σ*) is the sum of the interaction energies for each pair of neighbouring blocks in *σ*. The normalizing constant in (2.1) is assumed unknown. A *state* is any choice of coordinates for the blocks that satisfy the conditions of the CBA model. We can write

2.2 Ω=T×O,

where *T* is the state space and *O* is all choices of orientations for the blocks in *T*. For each *b* ∈ *T*, we can assign the probability

2.3 $$\mu (b)\propto \sum_{o\in O}e^{-\varepsilon (b,o)/k_{B}T_{0}},$$

where ε(b,o) is the sum of interaction energies for each pair of neighbouring blocks in the configuration (b,o)∈Ω. Accordingly, we can write

2.4 π(b,o)=μ(b)λ(o;b),

where $\lambda (o;b)\propto \exp(-\varepsilon (b,o)/k_{B}T_{0})$ is the probability of the blocks having orientations o∈O given that the blocks are arranged according to *b*.

## 3. Equivalence class sampling

We wish to estimate the stationary state properties of the CBA model by sampling states from *T* and orientations from *O* according to the target distributions μ(⋅) and λ(o;⋅), respectively. We will focus exclusively on the problem of sampling from *T*, because the problem of sampling orientations (which is equivalent to sampling the Ising model) has been studied thoroughly in other contexts [28].

### 3.1. State-space reduction and Markov chain Monte Carlo

The ECS technique involves two stages. In the first stage, the state space *T* is reduced in such a way that some ‘target information’ is preserved and the remaining superfluous information is eliminated. For the self-assembly problem, the target information pertains to the *kinds of islands* that are likely to appear according to (2.3). We, therefore, begin by clarifying the wording ‘kinds of islands’ in terms of the CBA model. Let *b* be an arbitrary state of the CBA model and denote the blocks in *b* by $t_{1},t_{2},\ldots ,t_{N}$. An *island* in *b* is a subset of blocks *I* from *b* such that

I.1. *I* cannot be split into two disjoint subsets *I*_1_ and *I*_2_ in which there is no pair *t*_*a*_ ∈ *I*_1_ and *t*_*a*_ ∈ *I*_2_ which are neighbours, and
I.2. There does not exist a block *t_*c*_* ∈ *I* such that *t_*c*_* ∈ *b* and *I′* = *I *∪ *t*_*c*_ satisfies I.1.

I.1. essentially says that an island cannot be split into two disjoint parts, and I.2. asserts than a subset of an island is not an island. Consider the two states *b*_1_ and *b*_2_ shown in figure 2*b.* Imprecisely, we can say that both *b*_1_ and *b*_2_ contain ‘same kinds’ of islands because if we rotate the islands in *b*_1_ and translate them about the lattice, we can achieve *b*_2_. More precisely, two states *b*_1_ and *b*_2_ are said to be *rotational isomorphs* if

RI.1. Each island in *b*_1_ can be mapped onto an island in *b*_2_ by rotations and translations alone, and
RI.2. this mapping is one-to-one and onto between the islands of *b*_1_ and the islands of *b*_2_.

Similarly, we say that two islands *I*_1_ and *I*_2_ are rotational isomorphs if *I*_1_ can be mapped onto *I*_2_ with rotations and translations alone. We can, therefore, partition *T* into *equivalence classes E*_1_, *E*_2_, … ,*E*_*h*_, where any two states *b*_1_ and *b*_2_ belong to the same equivalence class if and only if *b*_1_ and *b*_2_ are rotational isomorphs. We refer to the collection

3.1 $$H=\{E_{1},E_{2},\ldots ,E_{h}\},$$

as the *reduced state space*. We also have the following.

Theorem 3.1. *Let b*_1_ *and b*_2_ *be two states from the CBA model. If b*_1_ *and b*_2_ *belong to the same equivalence class, then μ*(*b*_1_) = *μ*(*b*_2_), *where μ is the probability measure in equation* (*2.3*).

Proof of theorem 3.1 is provided in appendix A.3. According to theorem 3.1, we can assign the probability

3.2 $$\upsilon (E_{k})\propto n(E_{k})\mu (E_{k})$$

to equivalence class *E*_*k*_ ∈ *H*, where *n*(*E*_*k*_) is the number of states that belong to equivalence class *E*_*k*_ (the *degeneracy* of *E*_*k*_), and *μ*(*E*_*k*_) is the probability from equation (2.3) calculated for any member of *E*_*k*_. The problem of ‘what kinds of islands appear at equilibrium’ can be stated as ‘which equivalence cases occur with relatively high probability *v*’. In order to obtain this target information, we only need to consider the reduced state space *H*, rather than the (considerably larger) space *T*. Note that, in contrast with the state space *T*, the reduced state space *H* does not grow as the dimension *d* of the lattice increases (providing that the number blocks *N* is fixed). Thus, for very large *d*, the reduced state space *H* will be considerably smaller than the full state space *T*.

In passing, we note an important physical aspect of the ECS approach. It can be shown that

3.3 $$\upsilon (E_{k})\propto e^{-A(E_{k})/k_{B}T_{0}},$$

where *A*(*E*_*k*_) can be regarded as the ‘free energy of equivalence class *E*_*k*_’. This is discussed in detail in appendix A.1. ECS, therefore, directly incorporates thermodynamics into the MCMC sampling framework, which is a highly desirable feature when the equilibrium (stationary) state properties of a molecular self-assembly model are of interest.

In order to implement ECS in a computational environment, it is necessary to use a *canonical representation* for each equivalence class in *H* (figure 2*c*). In this study, the canonical representation *C*_*k*_ of equivalence class *E*_*k*_ is generated by choosing an arbitrary state *b* from *E*_*k*_, and letting the elements of *C*_*k*_ be the islands from *b* as well as one empty element. The empty element is necessary for our implementation of MCMC as described in the following section. We do not distinguish between canonical representations that can be made equivalent by rotational isomorphism of their elements or re-ordering of their elements. Therefore, a canonical representation *C*_*k*_ is a unique representation of the equivalence class *E*_*k*_. The *number of islands* in equivalence class *E*_*k*_ is the number |*C*_*k*_| − 1.

### 3.2. Extension–reduction chain

To sample equivalence classes via the MCMC algorithm, we will employ the MH algorithm. We use *Z* to denote the Markov chain generated by the MH algorithm (the ‘MH chain’), and *Y* to denote the Markov chain used to generate proposals for the MH algorithm (the ‘proposal chain’). In this section, we introduce a suitable proposal chain called *extension–reduction chain*. To define the extension–reduction chain, we first construct a transformation *K* that maps equivalence classes to other equivalence classes at random. Fix an equivalence class *E*_*s*_ with canonical representation *C*_*s*_ and consider the following actions.

T.1. Choose island *k* from *C*_*s*_ with probability
  3.4 $$P_{k}=\frac{i_{k}^{\alpha_{1}}}{\sum_{h=0}^{\left|C_{s}\right|}i_{h}^{\alpha_{1}}},$$
  where $i_{k}$ is the number of blocks in island *k* and *α*_1_ is a positive, non-zero constant. Now, choose island *j* from *C*_*s*_ with probability
  3.5 $$Q_{jk}=\frac{1+i_{j}^{\alpha_{2}}}{\sum_{h=0}^{\left|C_{s}\right|}\left(1+i_{h}^{\alpha_{2}})-(1+i_{k}^{\alpha_{2}}\right)},$$
  where *α*_2_ is a positive, non-zero constant.
T.2. For the island *k* chosen in step T.1, let $I_{k}^{-}$ be the set of all islands that can be obtained by deleting a single block from island *k*. Each of the islands in $I_{k}^{-}$ must satisfy the conditions I.1 and I.2 outlined earlier. Now, if there are pairs of islands in $I_{k}^{-}$ that are rotationally isomorphic, delete one member of the pair and repeat until there are no pairs of islands in $I_{k}^{-}$ which are rotationally isomorphic. Denote the resulting set by $T_{k}^{-}$. $T_{k}^{-}$ is called the *reduction set* of island *k*.
T.3. For the island *j* chosen in step T.1, let $I_{j}^{+}$ be the set of all islands that can be obtained by adding a single block to island *j*. Then, delete islands from $I_{j}^{+}$ until there are no two pairs of islands in $I_{j}^{+}$ which are rotationally isomorphic. Denote the resulting set by $T_{j}^{+}$. $T_{j}^{+}$ is called the *extension set* of island *j*.
T.4. Replace island *k* in *C_s_* with a uniformly chosen element of $T_{k}^{-}$, and replace island *j* in *C_s_* with a uniformly chosen element of $T_{j}^{+}$. This produces a set $C_{s}^{\prime }$. Add one empty island to $C_{s}^{\prime }$ if there are no empty islands in $C_{s}^{\prime }$. If there are two empty islands in $C_{s}^{\prime }$, delete one empty island. Set *C*_*r*_ = $C_{s}^{\prime }$.

**Figure 3.**
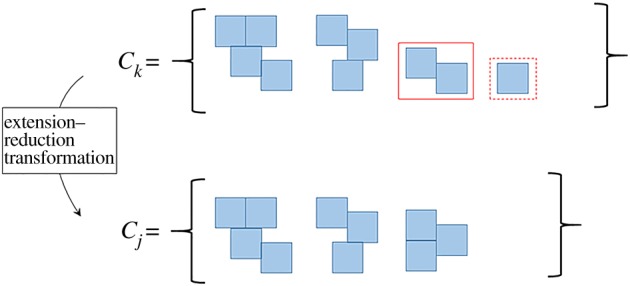
Example of an extension–reduction transformation on equivalence class *E*_*k*_. The island in the red box is extended and the island in the dotted red box is reduced.

The resulting set *C*_*r*_ is a canonical representation for an equivalence class *E*_*r*_ which may or may not be identical to *E*_*s*_. This transformation is denoted with the symbol *K*, and is referred to as the *extension–reduction* transformation. An example of an outcome of the extension–reduction transformation is shown in figure 3. Note that by virtue of equation (3.5), the empty island may be replaced with a single block with finite probability for any occurrence of the extension–reduction transformation. In such a case, we can intuitively think of the transformation as creating a new island by removing a block from an existing island.

The extension–reduction transformation *K* maps equivalence classes to equivalence classes at random. The probability

3.6 $$q^{k\to j}=P(K(C_{k})=C_{j})$$

is evaluated in appendix A.5. With this characterization of the extension–reduction transformation, we can generate a Markov chain with state space *H* as follows.

Theorem 3.2. *Choose an arbitrary equivalence class E_*j*_ from H and set Y*_1_ = *C_*j*_*, *Y*_2_ = *K*(*Y*_1_), *Y*_3_ = *K*(*Y*_2_), *and so on*. *Then the sequence Y*_1_, *Y*_2_, *Y*_3_, … *is a υ-irreducible and aperiodic Markov chain on H with transition probability* $P(Y_{2}=E_{j}|Y_{1}=E_{k})=q^{k\to j}$.

Theorem 3.2 is proved in appendix A.3. The Markov chain *Y* in theorem 3.2 is the *extension–reduction chain*. The parameters *α*_1_ and *α*_2_ in equations (3.4) and (3.5) can be used to control the evolution of the *Y*. *Y* does not have stationary distribution *υ*, and to perform ECS we therefore use the transition probability in equation (3.6) to simulate the standard MH chain *Z*.

### 3.3. Approximation for the degeneracy factor

It does not appear possible to provide closed-form expressions for the degeneracy factor *n*(*E*_*k*_) in equation (3.2) in the general case. However, we can form a good approximation to it with the following two-step method. (a) Compute the degeneracy factor exactly for a special case in which each island only covers a single lattice point. (b) Show that the degeneracy factor for the actual CBA model converges to the degeneracy factor obtained from this special case in a ‘low-coverage limit’, in which the lattice is extremely large compared with the number of blocks.

The concept of a *semicanonical representation* is needed to carry out step (a) described above. Choose an equivalence class *E*_*k*_ and let *b*_1_ and *b*_2_ be any two states that belong to *E*_*k*_. Let *U*_*k*_ and *V*_*k*_ be the set of islands belonging to *b*_1_ and *b*_2_, respectively. Suppose that *U*_*k*_ and *V*_*k*_ can be made equivalent by translation of their islands *without rotation*. *U*_*k*_ and *V*_*k*_ are called *semicanonical representations* of equivalence class *E*_*k*_. We do not distinguish between semicanonical representations that are equivalent under translation of islands, and therefore *U*_*k*_ and *V*_*k*_ are considered identical semicanonical representations. A canonical representation is also a semicanonical representation; however, two sets of islands corresponding to the same canonical representation may correspond to different semicanonical representations. Now, fix a semicanonical representation *V*_*k*_. We can partition the set of islands in *V*_*k*_ into subsets $V_{k}^{1}$, $V_{k}^{2}$, … , $V_{k}^{r}$, where two islands belong to the same subset if and only if they can be made equivalent by translation about the lattice. We will refer to the quantity

3.7 $$n(V_{k})=\frac{d^{2}!}{(d^{2}-m)!a_{1}!a_{2}!\cdots a_{r}!},$$

as the *degeneracy of the semicanonical representation V_*k*_*, where *m* is the number of islands contained in *V*_*k*_, and *a*_1_, … , *a*_*r*_ are the number of islands belonging to the subsets $V_{k}^{1}$, $V_{k}^{2}$, … , $V_{k}^{r}$ described above.

We now consider the meaning of *n*(*V*_*k*_) in equation (3.7). *n*(*V*_*k*_) is the number of ways to divide the *d*^2^ points of the torus **T**_*d*_ into *r* + 1 subsets $V_{k}^{0}$, $V_{k}^{1}$, $V_{k}^{2}$, … , $V_{k}^{r}$, where permutation of any points belonging to the same subset is does affect *n*(*V*_*k*_). The sets $V_{k}^{1}$, $V_{k}^{2}$, … , $V_{k}^{r}$ are exactly those defined in the previous paragraph, whereas the set $V_{k}^{0}$ can be thought of as the set of *d*^2^ – *m* ‘unoccupied’ points of the lattice. Equation (3.7) therefore counts the number of ways to assign islands from a semicanonical representation to the lattice, but in such a way that the islands only cover a single point of the lattice while retaining information on their shape. The number

3.8 $$n_{0}(E_{k})=\sum_{V\in \Gamma (E_{k})}n(V),$$

where *Γ*(*E*_*k*_) is the set of all distinct semicanonical representations available to equivalence class *E*_*k*_, can be interpreted as a degeneracy factor of the case where the islands only cover a single point of the lattice. Considering the actual degeneracy factor *n*(*E*_*k*_) in equation (3.2), we in general have that *n*(*E*_*k*_) < *n*_0_(*E*_*k*_). However, in appendix A.4, we show that for any equivalence class *E*_*k*_,

3.9 $$\frac{n(E_{k})}{n_{0}(E_{k})}\to 1,$$

as *d *→ ∞ with *N* (the number of blocks) fixed. Following step (b) mentioned above, we therefore employ the *low-coverage approximation*

3.10 $$n(E_{k})\approx n_{0}(E_{k}),$$

in order to perform ECS in the following sections. The low-coverage approximation is mathematically permissible for the following reason. Let *Z* and *Z*^0^ be the MH Markov chains when sampling from the actual distribution *υ* in equation (3.2) and when using the low-coverage approximation, respectively. Without loss, we can take *Z* and *Z*^0^ to be *υ*- and *υ*_0_(*E*_*k*_) = *n*_0_(*E*_*k*_)*μ*(*E*_*k*_)-distributed random variables, respectively. Equation (3.9) then implies that max_*E*__∈*H*_|*υ*_0_(*E*) − *υ*(*E*)| → 0 and hence that *Z* converges to *Z*^0^ in distribution as *d *→ ∞. From a physical point of view, the low-coverage approximation in equations (3.8) and (3.10) has connections with the statistical mechanical concept of indistinguishable and distinguishable particles. This suggests that under the low-coverage approximation, the CBA model might be regarded as a kind of ‘two-dimensional lattice gas’ model. This very natural physical picture further supports the application of the low-coverage approximation in ECS. Note that study of MCMC Markov chains with approximate transition kernels appears to related to research on the ‘pseudo marginal’ MCMC algorithm [29–32].

### 3.4. Computational implementation

**Figure 4.**
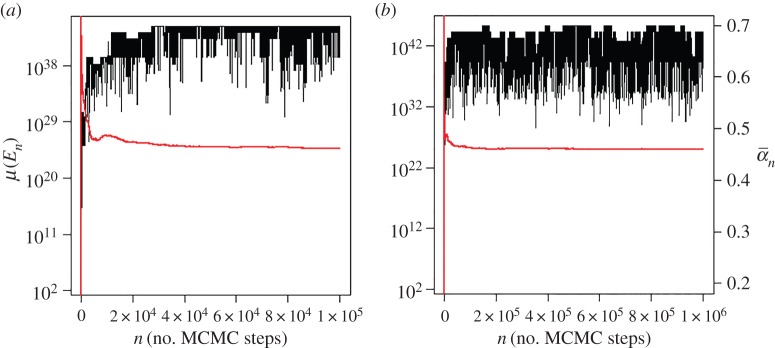
Plot of the quantity *μ* in equation (3.2) as a function of *Z*_*n*_, the state of the MH chain at step *n*, for a typical simulation of ECS at 100 K (see text for details). The red line corresponds to *a*_*n*_, the acceptance rates up to step *n*. Plot (*a*) shows the first 10^5^ steps of the simulation, and plot (*b*) shows the entire 10^6^ steps of the simulation.

We have written a code called *IslandPrediction* which performs ECS on the CBA model as described above. To enhance convergence rates, the *IslandPrediction* code incorporates parallel tempering into the MH algorithm, exactly as described in reference [33]. The replica MH chains differ in the temperature of their stationary distribution, and each MH Markov chain has proposals generated according to its own extension–reduction chain, where these extension–reduction chains are simulated independently of one another. *IslandPrediction* is available as an *R* script [34,35] and as a program written in *Go* [36] (both language codes available upon request). The *Go* version of *IslandPrediction* is intended for multicore environments. A striking feature of *IslandPrediction* is that the MH Markov chain converges relatively quickly with the length of the extension–reduction chain. Using the conditions reported in table 1, we observe burn-in periods of around 20 000–30 000 steps of the MH algorithm and an asymptotic acceptance rate of between 0.1 and 0.5 (figure 4). Reasonable estimates of quantities such as island size distribution can be obtained after 100 000 steps of the MH algorithm, assuming that the burn-in period has been neglected. The simulations presented in the following section were actually run for 10^6^ steps, with the first 5 × 10^5^ steps removed before analysis. Results acquired with the shorter simulation criterion of only 100 000 steps described above showed no significant difference from the longer simulations presented in the following section. Computation of the transition probabilities $q^{i\to j}$ in equations (A 25)–(A 28) is the major performance bottleneck in these calculations. These calculations can take between 0.5 to about 5 s depending on the number of and size of the islands in the canonical representations, and running the entire simulation for 100 000 steps of the MH algorithm typically takes around 16 h for the case of 10 blocks when the *R* code is used on a typical desktop computer. By contrast, simulations of 100 000 and 10^6^ steps of the MH algorithm only take around 30 min and 3 h, respectively, when the *Go* version of *IslandPrediction* is run on a 16 core cluster.

**Table 1.** Parameters used in the simulations described in §4. $T_{0}^{1}$, $T_{0}^{2}$ and $T_{0}^{\mathrm{step}}$, respectively, refer to the lowest temperature, highest temperature and temperature step used for parallel tempering.

$\varepsilon_{P,1,1}$	−0.1 eV	$\varepsilon_{P,1,2}$	0.03 eV
$\varepsilon_{S,1,1}$	−0.08 eV	$\varepsilon_{S,1,2}$	0.02 eV
$\varepsilon_{T,1,1}$	−0.05 eV	$\varepsilon_{T,1,2}$	−0.01 eV
$\varepsilon_{P,2,2}$	0.05 eV	*d*	50
$\varepsilon_{S,2,2}$	0.025 eV	*N*	10
$\varepsilon_{T,2,2}$	0.10 eV	$\alpha_{1}$, $\alpha_{2}$	1, 1
$T_{0}^{1}$	100 K	$T_{0}^{2}$	200 K
$T_{0}^{\mathrm{step}}$	10 K		

## 4. Simulation results

**Figure 5.**
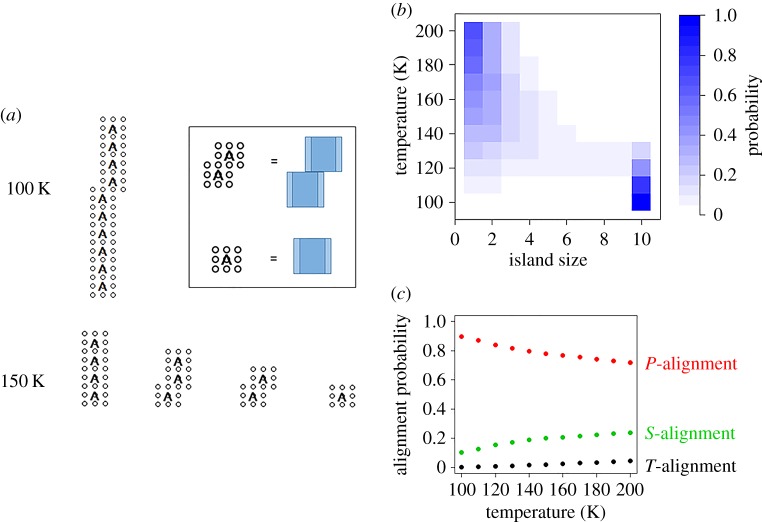
(*a*) An equivalences class randomly chosen beyond the burn-in period for the simulation performed at 100 K (top row), and for the simulation performed at 150 K (bottom row). The insert explains how the graphics should be interpreted. (*b*) Island size distribution estimated between 100 and 200 K (see text). (*c*) Alignment distribution estimated between 100 and 200 K (see text for details). The sample of equivalence classes used to generate this figure is available as the electronic supplementary material.

We now present results obtained using the ECS procedure with the conditions shown in table 1. The block orientations of any interesting islands were optimized individually using a standard technique used to study Ising models (see appendix A.1). Figure 5*a* shows randomly chosen equivalence classes that appeared beyond the burn-in regime in our calculations at 100 and 150 K. At low temperatures, we find that equivalence classes almost always contain a single island, whereas at higher temperatures, these equivalence classes instead consist of multiple, smaller islands. This observation is quantified in figure 5*b*, which shows the island size distributions computed from the above simulations. The island size distribution *f*_*k*_ is the probability of an island containing *k* blocks appearing in an equivalence class chosen randomly with respect to equation (3.2). At high temperatures, the island size distribution *f_*k*_* decays rapidly with the number of blocks *k* in the island. A similar decay was reported for the graphene size distribution obtained in the experiment described in fig. 1 [4], which suggests that in these experiments, graphene was produced under conditions in which many small islands are present.

The behaviour of the model is most interesting at low temperatures. As temperature decreases, the distribution spreads towards larger island sizes. At about 120 K, a second sharp mode starts to appear at *k* = 10 blocks. The occurrence of this mode shows that, at low temperatures, once every block gathers into a single island, the blocks tend to remain in this state. In order to move away from this state, one of the blocks in this island must break away, resulting in a state containing two islands. One of these islands contains nine blocks, and one contains one block. From a physical point of view, this second island contributes some entropy to the model; however, it also increases the overall energy of the model. This energy cost is particularly important at low temperature, and so this state either quickly reverts back to being a single large island, or another block departs from the island of size nine and joins the island containing one block. The appearance of two modes is therefore attributed to the fact that, once one block detaches from a large island, other blocks can be expected to detach in order to stabilize this block. As temperature decreases further, the blocks become increasingly stable when they are all part of a single island, and the probability density becomes concentrated on islands of size 10.

Figure 5*c* shows the alignment distribution estimated from the above simulations. This corresponds to the expected fraction of *P-, S-* or *T*-type alignments observed in a randomly chosen equivalence class. At very low temperatures, almost all alignments are of the *P*-type. This quantifies the observation that large, *chain-shaped* islands tend to appear at low temperature, in agreement with the experimental observations described in figure 1. As temperature increases, the fraction of *S*-type alignments increases. This is a consequence of the entropic contribution to the model (described by the degeneracy term in equation (3.8)), which favours the appearance of islands of low symmetry.

## 5. Conclusion

Large state-space problems are rapidly becoming commonplace in most fields of science. For these problems, it is essential to have available methods that can efficiently extract ‘target information’ without burdening the researcher with volumes of superfluous information. This paper has presented a simple molecular self-assembly model that contains the basic elements of a large state-space problem, and developed the ECS technique for predicting a particular piece of target information (the ‘kinds of islands’ that appear with high probability in the model) with high precision. The key feature of ECS is that the model state space is reduced to a set of equivalence classes, where two states *b*_1_ and *b*_2_ belong to the same equivalence class if and only if *b*_1_ can be transformed into *b*_2_ under a suitably chosen map. By development of a special Markov chain on this reduced state space, we achieved efficient estimation of the target information in the model via MCMC sampling. While we have considered the special (but important) example of molecular self-assembly, we believe that the approach used in this study provides a ‘line-of-thinking’ also for other kinds of large state-space problems.

Two interesting mathematical problems are encountered in the development of ECS. In order to generate proposals for the MCMC sampling component of ECS (assuming that the MH algorithm is employed), one needs a Markov chain with state space *H* and calculable transition probabilities, where *H* is the collection of equivalence classes (reduced state space). Moreover, good estimates of the quantities *n*(*E*_*k*_), the number of states contained in equivalence class *E*_*k*_, are needed to ensure that the MCMC algorithm samples from a distribution that is sufficiently close to the actual target distribution of the model. In this paper, we presented the extension–reduction chain as one example of such a Markov chain. However, there is motivation to develop other Markov chains and to study the convergence properties of the algorithms in detail. In particular, the types of Markov chains available for ECS are expected to depend upon the specific problem under consideration. We also presented an approximation for *n*(*E*_*k*_) in the case of low-coverage of molecules on the surface, and showed that this approximation converges as desired in the limit of zero surface coverage. While many experimental studies of molecular self-assembly use low-coverage conditions, it may also be interesting to develop other approximations to the degeneracies *n*(*E*_*k*_) that hold in the high-coverage limit. While not so relevant for the generation of small molecular islands, experiments in the high-coverage limit are widely used to generate so-called two-dimensional crystals via molecular self-assembly [24]. The high-coverage regime is also of academic interest as it often involves relatively unintuitive self-assembly behaviour [9]. Another open problem for implementing ECS algorithms concerns the development of efficient algorithms for detecting rotational isomorphism between islands and configurations of self-assembly models. This computation is a bottleneck in our current implementation of ECS. Perhaps, the greatest merit of studying molecular self-assembly via ECS is that the routes to improvement are very clear. We will explore them in detail in future research.

## Data accessibility

The equivalence class sample used to generate figure 5 is available as an .Rdata workspace file for *R*. Upon loading this data into the *R* environment, one should type ‘Message’ to receive an explanation of the data structure. This data can be freely downloaded at http://dx.doi.org/10.5061/dryad.vd156.

## Appendix A

**A.1. Equivalence class sampling and free energy**

Observe that equation (2.3) in the main text defines an ‘orientation partition function’ for the state *b*. From statistical thermodynamics, we therefore obtain the identity

A 1 $$A_{o}(b)=-k_{B}T\;\ln\;Z_{o}(b),$$

where $Z_{o}(b)=\mu (b)$, and $A_{o}(b)$ can be regarded as a ‘partial’ free energy. The energetic component of $A_{o}(b)$ arises from the interactions between neighbouring blocks in *b*, and the entropic component arises from the multiple ways in which orientations can be assigned to the blocks in *b*. Note that $A_{o}(b)$ will have the same value for all *b* in equivalence class $E_{k}$ (see appendix A.3), and so we can write $A_{o}(E_{k})=A_{o}(b)$ without any ambiguity. Now consider the ‘degeneracy factor’ $n(E_{k})$ in equation (3.2). We can define the entropy

A 2 $$S(E_{k})=k_{B}\log\;n(E_{k}),$$

which arises from the multiple ways to place the blocks on the lattice such that the resulting state is in equivalence class $E_{k}$. Solving equations (A 1) and (A 2) for $n(E_{k})$ and $Z_{o}(b)$, respectively, and substituting the results into equation (3.2) of the main text gives

A 3 $$\upsilon (E_{k})\propto e^{-A(E_{k})/k_{B}T},$$

where the ‘full’ free energy $A(E_{k})$ is defined as

A 4 $$A(E_{k})=A_{o}(E_{k})-TS(E_{k}).$$

In this free energy, the energetic component arises from the interaction between neighbouring blocks for each state *b* in equivalence class $E_{k}$, and the entropic component is a sum of two sources of entropy: an entropy arising from the multiple ways of choosing the orientations for the blocks, and an entropy arising from the multiple ways of placing the blocks on the lattice to achieve a state from equivalence class $E_{k}$.

In the view of equation (A 3), ECS chooses equivalence classes with respect to a free energy. This is in contrast with sampling the configurations directly from (2.1), in which configurations are chosen with respect to their energy.

**A.2. MCMC for sampling of block orientation distribution**

For an equivalence class *E* with canonical representation *C*, consider an island *I* from *C*. Let *O* be the orientation space for the blocks in *I*. For convenience, denote the possible two orientations of the blocks as *α* and *β*, respectively. A Markov chain *Y* on *O* can be generated as follows. We generate a sequence of random variables *Bi*_1_, *Bi*_2_, … , *Bi*_*m*_ from a binomial distribution with *n*_*I*_ trials and a probability *p* of success per trial, where *n*_*I*_ is the number of blocks in island *I*. Then, the *m*th step of the Markov chain, *Y*_*m*_ is generated by choosing *Bi*_*m*_ blocks at random and assigning them the orientation *β*, and assigning the orientation *α* to the other blocks. An MH algorithm using *Y* to generate proposals can then performed to analyse the distribution of the block orientations in the island *I*.

For all cases in this study, the MH algorithm appeared to converge within tens of seconds for each island analysed. Markov chains were typically run for 3000 steps with *p* = 0.5. The block orientations from *O* that appeared most often in the MH output were then identified as the optimum choice of block orientations for the island.

**A.3. Theorems and proofs**

Proof of theorem 3.1. Let us temporarily denote the two possible orientations for each block as *α* and *β*, respectively. We first consider the case where the states *b*_1_ and *b*_2_ each contain one island *I*_1_ and *I*_2_, respectively. If *b*_1_ and *b*_2_ are in the same equivalence class, then *I*_1_ and *I*_2_ are rotational isomorphs, and according to condition RI.1 of the main text there is a bijection *v* that maps the labels of the blocks in *I*_1_ to the labels of the blocks in *I*_2_. Let *m* be the number of times that the island *I*_1_ is rotated under the map *v*. Define the maps r:O→O and $f^{m}:O\to O$ such that for any $(o_{1},o_{2},\ldots ,o_{N})\in O$,

A 5 $$r(o_{1},o_{2},\ldots ,o_{N})=(o_{v(1)},o_{v(2)},\ldots ,o_{v(N)})$$

and

A 6 $$f^{m}(o_{1},o_{2},\ldots ,o_{N})=(f^{m}(o_{1}),\;f^{m}(o_{2}),\ldots ,f^{m}(o_{N})),$$

where

A 7 $$f^{m}(o_{k})=\left\{\begin{array}{ll} \alpha & m\;\mathrm{is}\;\mathrm{odd},\;o_{k}=\beta ,\\ \beta & m\;\mathrm{is}\;\mathrm{odd},\;o_{k}=\alpha ,\\ o_{k} & m\;\mathrm{is}\;\mathrm{even}. \end{array}\right.$$

Note that both r:O→O and $f^{m}:O\to O$ are bijective. For any $o=(o_{1},o_{2},\ldots ,o_{N})\in O,$ we have

A 8 $$\varepsilon (b_{1},o)=\varepsilon (b_{2},r\circ f^{m}(o)),$$

and hence

A 9 $$\sum_{o\in O}\left(e^{-c\varepsilon (b_{1},o)}-e^{-c\varepsilon (b_{2},r\circ f^{m}(o))}\right)=0,$$

where $c={\left(k_{B}T\right)}^{-1}$. However, we also have that $r\circ f^{m}:O\to O$ is bijective. This gives

A 10 $$\sum_{o\in O}e^{-c\varepsilon (b_{2},r\circ f^{m}(o))}=\sum_{o\in r\circ f^{m}(O)}e^{-c\varepsilon (b_{2},o)}=\sum_{o\in O}e^{-c\varepsilon (b_{2},o)},$$

where $r\circ f^{m}(O)={\left\{r\circ f^{m}(o)\right\}}_{o\in O}$. Substituting (A 10) into (A 9) and using the definition of *μ* in equation (2.3) of the main text gives $\mu (b_{1})=\mu (b_{2})$. We now consider the case where the states *b*_1_ and *b*_2_ are in the same equivalence class and contain islands $\left(I_{1},I_{2},\ldots ,I_{n}\right)$ and $\left(J_{1},J_{2},\ldots ,J_{n}\right)$, respectively. As can be seen from the definition in equation (2.3), we have

A 11 $$\mu (b_{1})=\mu (I_{1})\mu (I_{2})\cdots \mu (I_{n})$$

and

A 12 $$\mu (b_{2})=\mu (J_{1})\mu (J_{2})\cdots \mu (J_{n}).$$

According to condition RI.2 of the main text, there is a one-to-one correspondence between the islands in $\left(I_{1},I_{2},\ldots ,I_{n}\right)$ and $\left(J_{1},J_{2},\ldots ,J_{n}\right)$. Without loss, suppose that this correspondence is of the form $\left(I_{1},J_{1}\right)$, $(I_{2},J_{2}),\ldots ,$ and $\left(I_{n},J_{n}\right)$. Following the steps from the first part of the proof shows that $\mu (I_{k})=\mu (I_{k})$ for all *k*, which completes the proof. ▪

Proof of theorem 3.2. *Y* is a Markov chain, because the event that $Y_{n}=E_{k}$ is depends upon *Y_*n*_*_−1_ but not on *Y_*n*_*_−*m*_, for *n* ≥ *m* > 1. Moreover, by the definition of $q^{k\to j}$ we have that $P(Y_{2}=E_{j}|Y_{1}=E_{k})=q^{k\to j}$. We proceed as follows to prove that *Y* is υ-irreducible. Let *E*_*u*_ denote the equivalence class with a canonical representation

A 13 $$C_{u}=\{A_{1},A_{1},\ldots ,A_{1},\emptyset \},$$

where the island $A_{1}$, which is isomorphic to a single block, appears *N* times in (A 13). Now consider any arbitrary equivalence class $E_{k}$ with a canonical representation

A 14 $$C_{k}=\{I_{1},I_{2},\ldots ,I_{m},\emptyset \},$$

where the islands in $C_{k}$ are ordered such that $|I_{1}|\geq |I_{2}|\geq \cdots \geq |I_{m}|$, where $|I_{j}|$ is the number of blocks in island *I_j_*. Consider a transformation $E_{k}\to E_{j}$ where

A 15 $$C_{k}=\{I_{1},I_{2},\ldots ,I_{m},\emptyset \}$$

and

A 16 $$C_{j}=\{J_{1},I_{2},\ldots ,I_{m},A_{1},\emptyset \}$$

are the canonical representations of $E_{k}$ and $E_{j}$ and $|J_{1}|=|I_{1}|-1$. To describe this transformation, we say that island $I_{1}$ is *reduced* to *J*_1_ and that the empty island ∅ is *extended* to *A*_1_. This transformation occurs with probability,

A 17 $$q^{k\to j}\geq \frac{1}{|I_{1}^{-}|}P_{I_{1}}^{k}Q_{\emptyset ,I_{1}}^{k}>0,$$

where $P_{I_{1}}^{k}$ is the probability that island *I*_1_ of equivalence class *k* is reduced during the extension–reduction transformation, and $Q_{\emptyset ,I_{1}}^{k}$ is the probability that island ∅ is extended given that *I*_1_ is reduced during the extension–reduction transformation. These quantities correspond to equations (3.4) and (3.5) of the main text, respectively. We also have that the reverse transformation $E_{j}\to E_{k}$ occurs with probability

A 18 $$q^{j\to k}\geq \frac{1}{|J_{1}^{+}|}P_{A_{1}}^{j}Q_{J_{1},A_{1}}^{j}>0.$$

Applying this argument repeatedly shows that there is a finite sequence of extension–reduction transformations from an arbitrary equivalence class $E_{k}$ to $E_{u}$ with finite probability. We also have that there is a sequence of extension–reduction transformations from an arbitrary equivalence class $E_{h}$ to $E_{u}$ with finite probability Putting these arguments together shows that an arbitrary equivalence class $E_{k}$ can be transformed into another arbitrary equivalence class $E_{h}$ via a sequence of extension–reduction transformation with finite probability. This confirms the υ-irreducibility of the Markov chain *Y*. To prove the aperiodicity of *Y*, consider an equivalence class $E_{k}$ with canonical representation $C_{k}=\{I_{1},I_{2},\ldots ,A_{1},\emptyset \}$. By extending the empty island ∅ and reducing the island $A_{1}$, we can achieve the transformation $E_{k}\to E_{k}$. The event occurs with probability

A 19 $$q^{k\to k}\geq P_{A_{1}}^{k}Q_{\emptyset ,A_{1}}^{k}>0,$$

which shows that $\gcd(n-1:P(Y_{n}=E_{k}|Y_{1}=E_{k})>0)=1$. Aperiodicity for any arbitrary equivalence class then follows from the irreducibility of the Markov chain. ▪

**Remark.** A formula for the probability $q^{k\to j}$ is provided in appendix A.5.

**A.4. Proof of equation (3.9)**

Let **T**_*d*_ be a torus as constructed in §3.3 and fix an equivalence class *E*_*k*_. Suppose that *E*_*k*_ contains *m* islands. Let *x* and *y* be any distinct choice of *m* points from *X*_*d*_ _+_ _1_ and *Y*_*d*_ _+_ _1_, respectively. We call the set x×y a *grid*. We consider two families of grids, *F*_0_ and *F*_1_. Grids from *F*_0_ are generated without any restrictions on what points may be chosen from *X*_*d*_ _+_ _1_ and *Y_*d*_* _+_ _1_. The number of grids contained in *F*_0_ is equal to

A 20 $$s_{0}={\left(\begin{array}{c} d\\ m \end{array}\right)}^{2}.$$

Grids from *F*_1_ are generated as follows. Fix an integer *H* and choose *m* mutually exclusive sets of *H* consecutive integers *J*_1_, *J*_2_, … , *J*_*m*_ from the set {1, 2, … , *d*}. Choose another *m* mutually exclusive sets of *H* consecutive integers $J_{1}^{\prime }$, $J_{2}^{\prime }$, … , $J_{m}^{\prime }$ from the set {1, 2, … , *d*} independently of *J*_1_, *J*_2_, … , *J*_*m*_, and let

A 21 $$\left.\begin{array}{rl} x & =\overset{m}{\underset{k=1}{⋃}}\min(i\in J_{k})\\ \;\mathrm{and}\;y & =\overset{m}{\underset{k=1}{⋃}}\min(i\in J_{k}^{\prime }). \end{array}\right\}$$

The set x×y is therefore a grid. *F*_1_ is defined as the union of all grids which can be constructed in this manner, where the number *H* is identical for all such constructions. Letting *P*_*m*_ be the number of ways to choose *m* sets of *H* consecutive integers from the set {1, 2, … , *d*}, the number of grids contained in *F*_1_ is therefore

A 22 $$s_{1}=P_{m}^{2}.$$

This proof involves three steps. Step 1 involves computing the number *P*_*m*_ in equation (A 22). In step 2, we show that *s*_1_/*s*_0 _→ 1 as *d* → ∞ with *N* (number of blocks) fixed. In step 3, we show that for any equivalence class *E*_*k*_ containing *m* islands, *s*_1_/*s*_0_ → 1 implies that *n*(*E*_*k*_)/*n*_0_(*E*_*k*_) → 1, where *n*(*E*_*k*_) is the degeneracy factor for equivalence class *E*_*k*_, and *n*_0_(*E*_*k*_) is defined in equation (3.8).

*Step 1.* Let *J*_1_, *J*_2_, … , *J*_*m*_ be *m* mutually exclusive sets of *H* consecutive integers from the set {1, 2, … , *d*}. To compute *P*_*m*_, we adopt the convention that $J_{1}<J_{2}<\cdots <J_{m}$, where *J*_*a*_ < *J*_*b*_ means that there is no integer in *J*_*a*_ which is greater than or equal to any of the integers in *J_*b*_*. Let

A 23 $$Q=d-\underset{i\in J_{m}}{\max}\;i.$$

We claim that the largest value of *Q* occurs when $\underset{i\in J_{m}}{\max}\;i=mH$. Indeed, in this case we have $J_{1}=\{1,2,\ldots ,H\}$, $J_{2}=\{H+1,H+2,\ldots ,2H\},\ldots ,$ and

A 24 $$J_{m}=\{(m-1)H+1,(m-1)H+2,\ldots ,mH\}.$$

Let us now calculate the number of ways of choosing the sets *J*_1_, *J*_2_, … , *J*_*m*_ such that

A 25 $$\underset{i\in J_{m}}{\max}\;i=mH+q.$$

In this case, there must be a total of *q* integers in the set

A 26 $$\begin{array}{rl} S & =\left\{1,\ldots ,\underset{i\in J_{m}}{\max}i\right\}-\left\{{⋃}_{k=1}^{m}J_{k}\right\}\\ & ={⋃}_{j=1}^{m}K_{j}, \end{array}$$

where

A 27 $$K_{1}=\left\{1,\ldots ,\underset{i\in J_{1}}{\min}\;i-1\right\}$$

and

A 28 $$K_{j}=\left\{\underset{i\in J_{j-1}}{\max}\;i+1,\ldots ,\underset{i\in J_{j}}{\min}\;i-1\right\}.$$

If $\underset{i\in J_{1}}{\min}i=1$ we set $K_{1}=\emptyset$. If for any *j* ≤ *m* we have $\underset{i\in J_{j-1}}{\max}\;i=\underset{i\in J_{j}}{\min}\;i$, we set $K_{j}=\emptyset$. The number of ways of choosing *J*_1_, *J*_2_, … , *J*_*m*_ such that (A 25) is satisfied is therefore the number of ways of assigning *q* indistinguishable objects to the *m* ‘cells’ *K*_1_, *K*_2_, … , *K*_*m*_. This number is given by (p. 85 of [37])

A 29 $$P_{m}^{q}=\left(\begin{array}{c} q+m-1\\ q \end{array}\right).$$

The total number of ways of choosing sets of *H* consecutive integers *J*_1_, *J*_2_, … , *J*_*m*_ from the set {1,2, … , *d*} is then

A 30 $$P_{m}=\sum_{q=0}^{d-mH}P_{m}^{q}.$$

Substituting equation (A 30) into equation (A 22) yields a formula for *s*_1_.

*Step 2*. It is sufficient to show that

A 31 $$\sum_{q=0}^{d-B}\left(\begin{array}{c} q+A\\ q \end{array}\right)/\left(\begin{array}{c} d\\ m \end{array}\right)\to 1,$$

as *d *→ ∞, where *B* = *mH* and *A* = *m* – 1. Using the identities

A 32 $$\left(\begin{array}{c} q+A\\ q \end{array}\right)=\frac{(q+A)!}{q!A!}=\frac{(q+A)!}{A!((q+A)-A)!}=\left(\begin{array}{c} q+A\\ A \end{array}\right)$$

and

A 33 B=m(H−1)+1+A,

we can write

A 34 $$\sum_{q=0}^{d-B}\left(\begin{array}{c} q+A\\ q \end{array}\right)=\sum_{q=0}^{C-A}\left(\begin{array}{c} q+A\\ A \end{array}\right)=\left(\begin{array}{c} C+1\\ A+1 \end{array}\right)=\left(\begin{array}{c} C+1\\ m \end{array}\right),$$

where *C* = *d* – *m*(*H* – 1) – 1. Equation (A 34) was obtained with the diagonal summation identity (p. 104 of [37]). Now, for integer *R* > *m*, we have

A 35 $$\left(\begin{array}{c} R\\ m \end{array}\right)=\frac{1}{m!}R(R-1)\cdots (R-m+1).$$

Equations (A 34) and (A 35) then show that the limit in (A 31) holds if (*C* + 1)/*d *→ 1. This property is evident from the definition of *C*.

*Step 3*. Let *I*_1_, *I*_2_, … , *I*_*m*_ denote the islands contained in canonical representation *C*_*k*_ for equivalence class *E*_*k*_. For each 1 ≤ *k* ≤ *m*, choose one block *t*_*k*_ from *I*_*k*_. The blocks *t*_1_, *t*_2_, … , *t*_*m*_ are called *tagged blocks*. A state belonging to equivalence class *E*_*k*_ can be generated in the following way. Choose a grid *g* from *F*_0_, and then choose *m* points from *g*. Then, place the islands *I*_1_, *I*_2_, … , *I*_*m*_ on the torus **T**_*d*_ such that (a) the coordinates of the *m* tagged blocks have a one-to-one and onto correspondence with the *m* points chosen from *g* and (b) no two islands overlap. If condition (b) cannot be fulfilled, then we choose another grid from *F*_0_ and start again. We call this procedure *T*. We can also define anther procedure *T*_0_, which differs from *T* only in that condition (b) is not enforced. We can also define yet another procedure *T*_1_, which is identical to *T*_0_ except that the family *F*_0_ is replaced with the family *F*_1_.

Now, any state in *E*_*k*_ can be generated via *T*. The number of unique states (up to rotational isomorphism) that procedure *T* can yield is therefore equal to the degeneracy factor *n*(*E*_*k*_). Similarly, the number of unique states that procedure *T*_0_ can yield is equal to the quantity *n*_0_(*E*_*k*_) in equation (3.8) of the main text. Let *n*_1_(*E*_*k*_) be the number of unique states that procedure *T*_1_ can yield. For sufficiently large *H* and sufficiently large *d* > *mH*, we have

A 36 $$n_{1}(E_{k})<n(E_{k})<n_{0}(E_{k}).$$

It is sufficient to show that *s*_1_/*s*_0_* *→ 1 implies *n*_1_(*E*_*k*_)/*n*_0_(*E*_*k*_)* *→ 1. However, from the fact that *F*_1_ ⊂ *F*_0_, we can write the identity

A 37 $$F_{1}=F_{0}-F_{0}\setminus F_{1}.$$

*s*_1_/*s*_0_* *→ 1 then implies that $F_{0}\setminus F_{1}\to \emptyset$ and hence $F_{0}\to F_{1}$ as *d *→ ∞. However, according to procedures *T*_0_ and *T*_1_, the numbers *n*_1_(*E*_*k*_) only differ *n*_0_(*E*_*k*_) when the families *F*_1_ and *F*_0_ contain different grids. It therefore follows that *n*_1_(*E*_*k*_)/*n*_0_(*E*_*k*_)* *→ 1 and hence that *n*(*E*_*k*_)/*n*_0_(*E*_*k*_)* *→ 1 as *d *→ ∞. ▪

**A.5. Formulae for the probability *q*^*j*^^→^^*k*^**

In this section, we present a method to compute the probability *q*^*j→k*^ that the extension–reduction transformation will transform equivalence class *E*_*k*_ into equivalence class *E_*j*_*. Our calculation uses a *reaction representation* for the extension–reduction transformation *E*_*k*_ → *E*_*j*_. Let $R_{kj}^{\prime }=C_{k}\cup C_{j}$, where $C_{k}$ is a canonical representation of equivalence class $E_{k}$. Then, remove islands from $R_{kj}^{\prime }$ until $R_{kj}^{\prime }$ contains no pairs of rotational isomorphic islands, and denote the resulting set by $R_{kj}$. Label the islands in $R_{kj}$ as 1, 2, … , and *s*. For the *i*th element of $R_{kj}$, we define the numbers

A 38 $$n_{i}=\sum_{a\in C_{k}}1(a=R_{kj}(i))$$

and

A 39 $$m_{i}=\sum_{a\in C_{j}}1(a=R_{kj}(i)),$$

where $a=R_{kj}(i)$ is the event that island *a* and the *i*th island of $R_{kj}$ are rotational isomorphs, and 1(A) is the indicator function for event *A*. We define the *reactant set* for the transformation $E_{k}\to E_{j}$ as the vector

A 40 $$\mathrm{RC}T_{k\to j}=(n_{1},n_{2},\ldots ,n_{s}),$$

where *s* is the number of elements in the set $R_{kj}.$ Similarly, the *product set* for transformation $E_{k}\to E_{j}$ is defined as the vector

A 41 $$\mathrm{PD}T_{k\to j}=(m_{1},m_{2},\ldots ,m_{s}).$$

By convention, we set the *s*th element of $R_{kj}$ to be the empty island. This implies that $n_{s}=m_{s}=1$ for all extension–reduction transformations. The labels 1, 2, … , *s* correspond to unique elements of $R_{kj}.$ The *reaction characteristic* for the transformation $E_{k}\to E_{j}$ is the difference between the product and reactant sets,

A 42 $$\Delta_{k\to j}=\{\delta \in \mathrm{PD}T_{k\to j}-\mathrm{RC}T_{k\to j}:\delta \neq 0\}.$$

A *composition* is any realization of the reaction characteristic, where two compositions are considered equal if and only if they differ only in the ordering of their elements. A composition of the form {−1, −1, +1, +1} means that two islands *A* and *B* are lost and two new islands *C* and *B* are gained during the extension–reduction transformation $E_{k}\to E_{j}$. These islands can be uniquely identified by comparing the product set with the reactant set. The event that islands *A* and *B* are lost and islands *C* and *B* are gained can be expressed with the *reaction representation*

A 43 $$\left.\begin{array}{r} A\to C\\ B\to D. \end{array}\right\}$$

By convention, the island that gains a block is represented by the upper transformation A→C, and the island that loses a block is represented by the lower transformation B→D. We say that *A* is *extended* to *C* and *B* is *reduced* to *D* during this transformation. In analysing extension–reduction transformations with the composition {−1, −1, +1, +1}, we will also have to consider the case where *A* is reduced and *B* is extended, as well as the case where *C* is swapped for *D*.

It is therefore possible to characterize a particular extension–reduction transformation with the composition of its reaction characteristic, and we can speak of the *composition of an extension–reduction transformation* without ambiguity. The following theorem lists all possible compositions of the extension–reduction transformation.

Theorem. *There are exactly nine compositions for the extension–reduction transformation, as listed in* table 2.

**Table 2.** The nine possible compositions for the extension–reduction transformation. The compositions are referred to as ‘classes’. ∅ denotes the empty set.

class 1	{−1, −1, +1, +1}	class 6	{−1, +1, +1}
class 2	{−1, −1, +2}	class 7	{−1, +2}
class 3	{−1, −1, +1}	class 8	{−1, +1}
class 4	{−2, +1, +1}	class 9	∅
class 5	{−2, +1}		*Proof*. Without reference to any particular composition, consider a general reaction representation of the type

A 44 $$\left.\begin{array}{r} A\to C\\ B\to D. \end{array}\right\}$$

Unless explicitly mentioned, we will assume that none of *A*, *B*, *C* or *D* are empty islands. Let

A 45 M=2×1(A=D,B=C)+1(A=D,B≠C∨A≠D,B=C),

where A=D is the event that *A* and *D* are rotational isomorphs, and A≠D is the event that *A* and *B* are not rotational isomorphs. We have that *M* = 0, 1 or 2. For each of these three values of *M*, we have the possibilities that A≠B, A=B and ∅=A≠B, where ∅ denotes the empty island (B=∅ is excluded, because the empty island cannot be reduced). For each of these possibilities, we again have the possibilities that C≠D, C=D and C≠D=∅ (C=∅ is excluded, because the empty island cannot result from extension of an island). We analyse each of the 3 × 3 × 3 = 27 possibilities in tables 3–5. A reaction is said to be *realizable* if it is a possible occurrence of the extension–reduction transformation.

**Table 3.** Analysis of the *M* = 0 cases (see proof of the theorem in section A.5).

condition	reaction representation	realizable
$\begin{array}{l} A\neq B\\ C\neq D \end{array}$	$\begin{array}{l} A\to C\\ B\to D \end{array}$	yes
$\begin{array}{l} A\neq B\\ C=D \end{array}$	$\begin{array}{l} A\to C\\ B\to C \end{array}$	yes
$\begin{array}{l} A\neq B\\ C\neq D=\emptyset \end{array}$	$\begin{array}{l} A\to C\\ B\to \emptyset \end{array}$	yes
$\begin{array}{l} A=B\\ C\neq D \end{array}$	$\begin{array}{l} A\to C\\ A\to D \end{array}$	yes
$\begin{array}{l} A=B\\ C=D \end{array}$	$\begin{array}{l} A\to C\\ A\to C \end{array}$	no^a^
$\begin{array}{l} A=B\\ C\neq D=\emptyset \end{array}$	$\begin{array}{l} A\to C\\ A\to \emptyset \end{array}$	yes
$\begin{array}{l} \emptyset =A\neq B\\ C\neq D \end{array}$	$\begin{array}{l} \emptyset \to C\\ B\to D \end{array}$	yes
$\begin{array}{l} \emptyset =A\neq B\\ C=D \end{array}$	$\begin{array}{l} \emptyset \to C\\ B\to C \end{array}$	yes
$\begin{array}{l} \emptyset =A\neq B\\ C\neq D=\emptyset \end{array}$	$\begin{array}{l} \emptyset \to C\\ B\to \emptyset \end{array}$	no^b^

^a^*C* cannot be in both the extension set and reduction set of *A*.

^b^B→∅ and C→∅ imply that both *B* and *C* contain only one block, and hence that *B* and *C* are rotational isomorphs. But this violates the condition M=0.

**Table 4.** Analysis of the *M* = 1 cases (see proof of the theorem in section A.5). We consider the two subcases C=B and D=A. For the case C=B, all cases with C=D or *A* = *B* are not realizable, as the former implies that *B* is reduced to *B*, and the latter implies that *B* is extended to *B*. For similar reasons, the cases C=D and A=B are not realizable when *D* = *A*.

condition	reaction representation	realizable
$\begin{array}{l} C=B\\ A\neq B\\ C\neq D \end{array}$	$\begin{array}{l} A\to B\\ B\to D \end{array}$	yes
$\begin{array}{l} C=B\\ A\neq B\\ C\neq D=\emptyset \end{array}$	$\begin{array}{l} A\to B\\ B\to \emptyset \end{array}$	no^a^
$\begin{array}{l} C=B\\ \emptyset =A\neq B\\ C\neq D \end{array}$	$\begin{array}{l} \emptyset \to B\\ B\to D \end{array}$	no^b^
$\begin{array}{l} C=B\\ \emptyset =A\neq B\\ C\neq D=\emptyset \end{array}$	$\begin{array}{l} \emptyset \to B\\ B\to \emptyset \end{array}$	yes
$\begin{array}{l} D=A\\ A\neq B\\ C\neq D \end{array}$	$\begin{array}{l} A\to C\\ B\to A \end{array}$	yes
$\begin{array}{l} D=A\\ A\neq B\\ C\neq D=\emptyset \end{array}$	$\begin{array}{l} A\to C\\ B\to \emptyset \end{array}$	no^c^
$\begin{array}{l} D=A\\ \emptyset =A\neq B\\ C\neq D \end{array}$	$\begin{array}{l} \emptyset \to C\\ B\to \emptyset \end{array}$	no^d^
$\begin{array}{l} D=A\\ \emptyset =A\neq B\\ C\neq D=\emptyset \end{array}$	$\begin{array}{l} \emptyset \to C\\ B\to \emptyset \end{array}$	no^d^

^a^If *B* is reduced to the empty island, the *B* must contain one block only, which incorrectly implies that A=∅.

^b^Extension of the empty island to *B* implies that *B* contains one block only. Reduction of *B* to *D* then incorrectly implies that *D* is an empty island.

^c^D=∅ and D=A incorrectly implies that A=∅.

^d^Reduction of *B* to the empty island, and extension of the empty island to *C* imply that both *B* and *C* contain a single block, and hence that B=C. But this violates the condition M=1.

**Table 5.** Analysis of the *M* = 2 case (see proof of the theorem in section A.5).

condition	reaction representation	realizable
A≠B	$\begin{array}{l} A\to B\\ B\to A \end{array}$	yes Following the analysis in tables 3–5, we can deduce the possible compositions for the reaction characteristic by considering each realizable reaction representation shown in the above tables. For example, the three reactions

$$\begin{array}{l} A\to C\;A\to C\;A\to C\\ B\to D\;B\to C\;B\to \emptyset , \end{array}$$

contribute compositions {−1, −1, +1, +1}, {−1, −1, +2} and {−1, −1, +1}, respectively, to the reaction characteristic. Proceeding in this manner yields the compositions listed in table 2. ▪

Formulae for $q^{k\to j}$ can be easily deduced by analysing each possible composition of the reaction characteristic case by case. For example, suppose that the reaction characteristic for extension–reduction transformation $E_{k}\to E_{j}$ has composition {−1, −1, +1, +1}. Tables 3–5 from the proof of theorem A.4 then show that this composition corresponds uniquely to the reaction representation

A 46 $$\begin{array}{l} A\to C\\ B\to D. \end{array}$$

The islands *A, B, C* and *D* can be identified by comparing the sets $\mathrm{RC}T_{k\to j}$ and $\mathrm{PD}T_{k\to j}$. The corresponding probability for the transformation $E_{k}\to E_{j}$ is

A 47 $$q^{k\to j}=n_{A}n_{B}(q_{BA,DC}^{k\to j}+q_{AB,DC}^{k\to j}+q_{BA,CD}^{k\to j}+q_{AB,CD}^{k\to j}),$$

where $n_{A}$ is the coefficient from $\mathrm{RC}T_{k\to j}$ which corresponds to island *A*, $q_{BA,DC}^{k\to j}$ is the probability that island *B* is reduced, island *A* is extended, island *D* is chosen from the reduction set of *B*, and island *C* is chosen from the extension set of *A*. According to §2 of the main text (particularly steps T.1–T.4 of the extension–reduction transformation), we have

A 48 $$q_{BA,DC}^{k\to j}=\left\{\begin{array}{ll} \frac{P_{B}^{k}Q_{AB}^{k}}{I_{B}^{-}I_{A}^{+}} & D\in I_{B}^{-},C\in I_{A}^{+}\\ 0 & \mathrm{otherwise} \end{array}\right.$$

where $P_{B}^{k}$ is the probability of reducing island *B* (equation (3.4) of the main text), $Q_{AB}^{k}$ is the probability of extending island *A* following reduction of island *B* (equation (3.5) of the main text), $I_{B}^{-}$ is the reduction set of *B*, and $I_{A}^{+}$ is the extension set of *A*. The notation $D\in I_{B}^{-}$ means that there is an island contained in $I_{B}^{-}$ that is rotationally isomorphic to *D*. The transition probabilities for every case in table 2 are listed in table 6.

**Table 6.** Transition probability for every possible composition of the extension–reduction transformation. Note that both reactions in class 8 contribute the same probability to the extension–reduction transformation.

class 1 {−1, −1, +1, +1}	
$\begin{array}{l} A\to C\\ B\to D \end{array}$	$q^{k\to j}=n_{A}n_{B}\left(q_{BA,DC}^{k\to j}+q_{AB,DC}^{k\to j}+q_{BA,CD}^{k\to j}+q_{AB,CD}^{k\to j}\right)$
class 2 {−1, −1, +2}	
$\begin{array}{l} A\to C\\ B\to C \end{array}$	$q^{k\to j}=n_{A}n_{B}\left(q_{BA,CC}^{k\to j}+q_{AB,CC}^{k\to j}\right)$
class 3 {−1, −1, +1}	
$\begin{array}{l} A\to C\\ B\to \emptyset \end{array}$	$q^{k\to j}=n_{A}n_{B}\left(q_{BA,\emptyset C}^{k\to j}+q_{AB,\emptyset C}^{k\to j}\right)$
class 4 {−2, +1, +1}	
$\begin{array}{l} A\to C\\ A\to D \end{array}$	$q^{k\to j}=n_{A}\left(n_{A}-1\right)\left(q_{AA,CD}^{k\to j}+q_{AA,DC}^{k\to j}\right)$
class 5 {−2, +1}	
$\begin{array}{l} A\to C\\ A\to \emptyset \end{array}$	$q^{k\to j}=n_{A}\left(n_{A}-1\right)q_{AA,\emptyset C}^{k\to j}$
class 6 {−1, −1, +1}	
$\begin{array}{l} \emptyset \to C\\ B\to D \end{array}$	$q^{k\to j}=n_{B}\left(q_{B\emptyset ,DC}^{k\to j}+q_{B\emptyset ,CD}^{k\to j}\right)$
class 7 {−1, +2}	
$\begin{array}{l} \emptyset \to C\\ B\to C \end{array}$	$q^{k\to j}=n_{B}q_{B\emptyset ,CC}^{k\to j}$
class 8 {−1, +1}	
$\begin{array}{l} A\to B\\ B\to D \end{array}$	$q^{k\to j}=\sum_{B\in R_{kj},B\neq A}n_{A}n_{B}\left(q_{BA,DB}^{k\to j}+q_{AB,BD}^{k\to j}\right)$
$\begin{array}{l} A\to C\\ B\to A \end{array}$	
class 9 {Ø}	
$\begin{array}{l} \emptyset \to B\\ B\to \emptyset \end{array}$	$q^{k\to j}=\sum_{B\in R_{kj}}n_{B}q_{B\emptyset ,\emptyset B}^{k\to j}+\sum_{A,B\in R_{kj}:A\neq B}n_{A}n_{B}\left(q_{AB,BA}^{k\to j}+q_{BA,BA}^{k\to j}\right)$
$\begin{array}{l} A\to B\\ B\to A \end{array}$	
